# The Nurse or Midwife at the Crossroads of Caring for Patients With Suicidal and Rigid Religious Ideations in Africa

**DOI:** 10.3389/fpsyg.2021.549766

**Published:** 2021-04-27

**Authors:** Lydia Aziato, Joyce B. P. Pwavra, Yennuten Paarima, Kennedy Dodam Konlan

**Affiliations:** ^1^School of Nursing and Midwifery, University of Ghana, Accra, Ghana; ^2^Department of Maternal and Child Health, School of Nursing and Midwifery, University of Ghana, Accra, Ghana; ^3^Department of Research, Education, and Administration, School of Nursing and Midwifery, University of Ghana, Accra, Ghana; ^4^Department of Adult Health, School of Nursing and Midwifery, University of Ghana, Accra, Ghana

**Keywords:** Africa, care, midwife, nurse, patient, religious, spiritual, suicide

## Abstract

Nurses and midwives are the majority of healthcare professionals globally, including Africa, and they provide care at all levels of the health system including community levels. Nurses and midwives contribute to the care of patients with rigid or dogmatic religious beliefs or those with suicidal ideations. This review paper discusses acute and chronic diseases that have suicidal tendencies such as terminal cancer, diseases with excruciating pain, physical disability, stroke, end-stage renal failure, and diabetics who are amputated. It was reiterated that nurses and midwives taking care of these patients should be alert and observant to identify their suicidal tendencies. The paper also discusses religious or spiritual inclinations that negatively affect healthcare access and adherence, especially to biomedical or western medicine. It was emphasized that some religious beliefs do not allow their followers to employ biomedical treatment and nurses and midwives should not impose their faith on patients and their families. The paper ends with a discussion on the specific roles of nurses and midwives in the care of patients with suicidal ideations such as assessment, counseling, administering medication, observation, social interaction, ensuring safety measures, and providing an enabling environment for the family to part of the care and for the observation of religious coping strategies. Nurses and midwives should enhance their knowledge and skills on suicide and increase public education on suicide prevention and identification of those at risk.

## Introduction

Nurses and midwives are the majority of healthcare professionals globally (Asamani et al., 2019). Nurses take care of many patients with suicidal ideations and varying spiritual orientations (Acheampong and Aziato, 2018). In Africa especially, some patients have spiritual ideas that negatively affect their health-seeking behavior and adherence to western treatment (Nardi and Rooda, 2011). Spiritual practices have been a component of healing within the African domain for many years before the introduction of western medicine (Asamoah-Gyadu, 2014). There is evidence that patients in Africa attribute the causes of health challenges to spiritual influences and employ spirituality in the healing of diseases (Ha et al., 2014). With the acceptance of religious leaders or activities within the western healthcare context in Africa (Asamoah-Gyadu, 2014), it presupposes that spirituality and its accompanying rituals or activities are to be incorporated into care.

Again, debilitating health challenges pose both psychological and physical effects that lead to suicidal ideations (Weaver, 2015). In the environment of psychiatric hospitals, interventions for suicidal patients are well delineated; but this is not the same in the acute setting, where patients also have suicidal tendencies (Goldman et al., 2015). The stigmatization associated with mental illness (Haw et al., 2013) could lead to patients with psychological problems having suicidal ideations attend general hospitals, and it is possible that known patients with mental illness having co-morbid conditions are admitted to acute care settings. Therefore, nurses and midwives in acute settings should have adequate knowledge and skills to assess patients to identify those with suicidal tendencies.

Nurses and midwives are mandated to respect the rights of patients and their families (Adinkrah, 2014) and to maintain confidentiality (Konlan et al., 2020). The patients are therefore not forced to take any treatment prescribed (Adinkrah, 2014). It is in this context that the nurse is in a dilemma regarding the respect for choices of patients and the scientific or medical benefits of the treatment regime recommended. This paper examines the health challenges that predispose to suicidal ideations, the dynamics of spirituality within healthcare settings, and the role of nurses and midwives in identifying and caring for patients with suicidal tendencies.

## Materials and Methods

This review paper sought literature from databases, such as Web of Science, PubMed, PsycINFO, and EBSCO, and the keywords used to search the literature-included care, suicide, religion, suicidal ideations, diseases, patients, and Africa. The authors retrieved data from the literature based on its relevance for the review. The focus of the review was to understand which acute and chronic diseases have suicidal tendencies, which specific religious beliefs in Africa interfere with healthcare access and adherence, and what roles nurses and midwives play in caring for patients with suicidal ideations. The authors discussed the data retrieved and applied their experiences as nurses in Ghana to provide discussions that are useful for nurses and midwives in Africa and globally since the issues of suicide and religion have global connotations. Subsequent sessions discussed the thematic areas for the review.

## Acute and Chronic Health Challenges with Suicidal Tendencies

This section focuses on various categories of patients with acute and chronic health challenges who have suicidal ideations such as those with terminal cancers, patients with disabilities, diabetics with amputation, and end-stage renal disease patients. Nurses are an influential cadre of health staff (Muller, 2000) and are pivotal at reversing the rising global burden of diseases (Sinding, 2003). There is a consensus on the significance of nursing interventions in improving patient outcomes and safeguarding patient safety (Muller, 2000; Sinding, 2003). In rendering nursing care, professional nurses and midwives in acute care settings could encounter patients harboring suicidal ideations (Bonsu et al., 2014) ranging from those with chronic diseases to those with psychiatric problems as well as patients with permanent disabilities.

One of the categories of patients who have been found to harbor suicidal ideations and should capture the attention of nurses are terminally ill breast cancer patients experiencing severe pain due to their conditions (Bonsu et al., 2014). Breast cancer is a devastating disease among women, and it is known to be associated with severe emotional and psychosocial consequences (Clegg-lamptey, 2007) including the ideas of suicide due to the excruciating pain at an advanced stage. In the study by Bonsu et al. (2014), it was identified that terminal breast cancer is associated with pain and that the pain is severe to the extent that some of the patients had suicidal ideations. Similar findings were found in the study by Ohene-Yeboah and Adjei (2012). Other studies (Doumit et al., 2007; Gauthier et al., 2009) also identified that the experience of excruciating pain in advanced breast cancer patients led to suicidal ideations among patients and that nurses ought to be quick in identifying these high-risk patients and provide appropriate care. This finding presupposes that patients with other pain associated conditions and other types of terminal cancer could have suicidal ideations (Shim and Hahm, 2011; Aziato and Adejumo, 2014). Thus, within the acute care setting, healthcare providers should be vigilant and provide the needed safety measures when such patients are being cared for.

Further, amputations and other associated disabilities were found to be associated with suicidal ideations (Turner et al., 2015). The study by Turner et al. (2015) concluded that suicidal ideations are common among those with recent amputations and nurses as well as other health team members ought to recognize this increased risk to prevent suicide. Also, suicide has been found to be higher among adults with disabilities compared to their counterparts without disabilities, and suicide is mostly preceded with suicidal ideations (Shim and Hahm, 2011; Acheampong and Aziato, 2018). It has further been observed that mothers with children having disabilities contemplated killing the children to make them free from the stigma of the disability (Fassberg et al., 2014). It was found that suicidal thoughts could linger in a person for several years, and this means health professionals ought to critically observe patients with disabilities as well as mothers with disable children over a long period of time if suicide is to be prevented (Shim and Hahm, 2011; Acheampong and Aziato, 2018). The literature thus recommends that nurses and other care practitioners involved in the care of the disabled are to recognize that these suicidal thoughts are precipitated mainly by discrimination (Acheampong and Aziato, 2018) and that there is the need for nurses to perform comprehensive assessments to identify these suicidal tendencies and arrange for appropriate interventions. Other studies (Shim and Hahm, 2011; Fassberg et al., 2014; McConnell et al., 2015; Acheampong and Aziato, 2018) have observed that adults with disabilities have higher rates of suicidal ideations compared to those without disabilities such as those who are blind. Blindness has been identified as a risk factor in suicidality (Lam et al., 2008). Thus, nurses caring for patients who are blind ought to be alert of this tendency and be proactive in their care.

In addition, patients with terminal chronic illness like End Stage Renal Diseases (ESRD; Hill and Pettit, 2013) are said to harbor suicidal ideations (Yamauchi et al., 2014). Hill and Pettit (2013) observed the dependence associated with dialysis could cause patients to harbor intentions of suicide since they lose their self-worth and are over-burdened with financial demands. The death by suicide in ESRD patients is 15 times that of the general population (Patel et al., 2012). There is a full range of possible suicidal behaviors that may be exhibited among those living with ESRD (Pompili et al., 2013). Some of the suicidal behaviors include thoughts of suicide, expressions of intent to attempt suicide (i.e., verbal threats or nonverbal actions such as giving away precious/valued items), developing a specific suicide plan (when, where, and how), and making a suicide attempt (Patel et al., 2012). In their work, Piscopo et al. (2016) observed that nurses ought to recognize that people living with ESRD may have pre-existing suicide risk factors, which may be activated and aggravated by the onset of chronic kidney disease. Such pre-existing risk factors may include mental illness, lack of social support, marital and family conflict, unemployment, financial and housing issues, other health concerns, and perception of the burden of illness (Pompili et al., 2013).

Also, stroke patients have suicidal tendencies (Bartoli et al., 2017). Nurses are to recognize that stroke patients might exhibit high intolerance of their disability, and previous studies have revealed higher rates of suicide ideation, suicide attempts, and completed suicides in post-stroke patients than in the general population (Bartoli et al., 2017). In different countries, cultural heritage and socio-economic status could influence a patient with a disability such as those associated with stroke to contemplate suicide (Eriksson et al., 2015). Post-stroke patients may feel like they have no hope, are a burden on others, or are losing their personal dignity and thus may have a desire for death (Bartoli et al., 2017) and nurses ought to recognize this key psychological challenge.

## Spirituality and Its Influence of Healthcare Access and Care

In the contemporary African hospital system, the religious needs of patients are acknowledged, and some avenues exist for patients and families to practice their faith (Nukunya, 2003). Religious beliefs play a critical role in survival, coping and maintaining the overall well-being within communities and many cultures in Africa especially when one is diagnosed with a chronic condition (Arrey et al., 2016). Religious beliefs are the institutionalized system or personal set of beliefs, attitudes, and practices; and, they are not what people do on Fridays in the mosque on Sundays in the church but a way of life. In Africa, religious beliefs operate at every level of society and play a critical role in health-seeking behaviors and adherence to treatment (Anarfi et al., 2016). Religious beliefs and practices are interconnected with spirituality. As such, some traditional practices to cure diseases are linked to spirituality in Africa (Alling, 2015).

Africa with an estimated population of about 1.2 billion people is home to religious sets such as Christianity, Islam, African traditional religions, and among others (Arrey et al., 2016). While modern medicine sees sickness to be an invasion of the human system by germs, most of these religious sets in Africa attribute sickness to evil spirits or the will of God (Onongha, 2015). This negatively affects their health-seeking behavior and adherence to western treatment (Alling, 2015). For instance, in a study among 523 diabetes patients of Islamic faith in Libya, participants attributed their illness to the will of God. They resorted to prayers, which greatly affected their adherence to western treatment (Ashur et al., 2015). In this regard, the nurse or midwife caring for patients with such religious inclination may have challenges administering their prescribed medications (Alling, 2015).

In Ghana, Anarfi et al. (2016) found that people who belong to the Pentecostal/Charismatic churches patronize prayer camp and faith healers, because they attribute sickness to evil spirits and therefore western medicines are not effective in treating illness perceived to be the cause of evil spirits. Again, people of the Roman Catholic faith prefer to visit their priest for prayers before accepting any western treatment. Besides, some patients of the Catholic faith will often pray “Hail Mary” before they resort to any western treatment (Adugbire and Aziato, 2020). In view of this, the nurse or midwife should provide avenues for patients to observe their spiritual beliefs in hospitals. The provision of such holistic care could enhance the satisfaction of the patient (Anarfi et al., 2016).

Runum (2014) in Nigeria reported that the Baha’i faith, which is rooted in Babism, believes humans are placed on earth to grow and develop spiritually. The Baha’i perceives illness as a factor for growth and should be approached both spiritually and materially. A Baha’i believes prayers are powerful and can cure illness. However, they have no objection to western medicine. They see prayers and western medicine as different aspects of the healing process God gave to mankind. The Baha’i permits the use of narcotics if ordered by the medical officer to control pain (Runum, 2014). In this instance, a nurse or midwife caring for patients from this faith would receive cooperation during the administration of treatment.

Similarly, individuals who belong to the African traditional religion belief sickness are transmitted by a malicious spirit, evil powers, ancestors, enemies, and witchcraft. For example, cancer, HIV/AIDS and diabetes, and other chronic conditions are considered by the African traditionalist as an ancestral curse or an invention of “mischievous” white persons (Onongha, 2015) and as such cannot be cured or treated with western medicines. This has affected the health-seeking behavior of the many African traditionalists as they seek supernatural and superstitious solutions to these medical conditions (Azongo and Yidana, 2015). This perception has been a great obstacle to the improvement of the living and health conditions among many African traditional religious sets. The traditional beliefs also interfere with adherence and may lead to late reporting of diseases to the hospital such as breast cancer (Clegg-lamptey, 2007). Those who report to the hospital may use some religious artifacts that nurses and midwives may not approve of or maybe afraid of (Anarfi et al., 2016). The artifacts may also interfere with care (Azongo and Yidana, 2015) depending on the body part applied.

Furthermore, in northern Ghana, people from indigenous traditional communities’ resort to consulting diviners who they consider as custodians of supernatural powers to cure their illness, which is often attributed to witchcraft and bad spirits. This perception affects their adherence to western medicine (Azongo and Yidana, 2015). Similarly, in Nigeria, the Yoruba belief that some people are from the spirit world will die at will (Abubakar et al., 2013). This belief is commonly referred to as the “Abiku.” With no particular signs and symptoms, diagnosing “abiku” is largely based on the individual’s non-responsiveness to biomedical treatment (Ogunjuyigbe, 2004). Based on this dogmatic belief, the nurse taking care of such a patient could experience stress and frustration if all treatment administered is not eliciting the expected outcome.

It is worth noting that socio-cultural beliefs in Africa also lead to poor patronage of western medicine. For example, it is acknowledged that the risk of being perceived as a “failed wife or woman” in society is a major determinant of women’s refusal to seek biomedical treatment for conditions that will require removal of the uterus or breast (Ugwu and De Kok, 2015). Also, the decision maker’s role in which the father/husband has the ultimate power to decide as to where and when a child or wife seeks treatment is a key socio-cultural issue in Africa. Fathers/husbands take the ultimate decision not only based on their figurehead role of the family but also due to their role in the provision of money for treatment (Abubakar et al., 2013). Thus, these socio-cultural orientations are interconnected with religiosity in Africa that negatively influence healthcare access and adherence. The situation is made worse with high poverty and dependency in Africa (Tang and Qin, 2015), so the real decision for not approving hospital care could be more than religiosity especially when hospital treatment is deemed expensive (Ugwu and De Kok, 2015) and the attitude of some healthcare professionals is also negative toward the poor patient (Konlan et al., 2020). It is therefore likely that the lack of use of biomedical treatment could lead to the worsening of a disease and that contributes to suicidal ideations. The nurse or midwife working within an environment with these beliefs and practices will be at crossroads in implementing biomedical health programs and interventions.

## The Role of the Nurse in the Care of Patients with Suicidal Ideations

The nurse or midwife has a significant role in the assessment, identification, management, and prevention of suicide by employing both systems and patient-level interventions (Davis et al., 2014) At the systems level, the nurse evaluates and controls environmental safety, improves protocols, policies, and practices consistent with zero suicide and assists in training for all categories of staff (Cureton and Elysia, 2015). At the patient level, the nurse assesses the consequences of all interventions, evaluates risk for suicide, monitors, and manages at-risk patients and provides suicide-specific psychotherapeutic interventions (Cutcliffe and Stevenson, 2007). In addition, nurses’ hands-on patient care is vital by taking all threats or suicide attempts seriously and highlighting a relationship agreement by developing rapport with the patient (Zaorsky et al., 2019).

There are several warning signs of suicidal ideation the nurse or midwife must pay attention to include observation of the patient looking for ways to kill him or herself, such as seeking access to pills, weapons, or other means of harm, exhibiting anger or revenge-seeking behavior, voicing hopelessness, and acting recklessly or engaging in risky activities (Talseth and Gilje, 2011). Other pointers that nurse or midwife should look out for are voicing feeling trapped, increasing alcohol or drug use, withdrawal from friends, family, and social activities, being anxious or agitated, having sleep disturbances or sleeping all the time, and having dramatic mood or personality changes (Talseth and Gilje, 2011). The nurse or midwife should be vigilant to identify patients at risk of suicide who talk or write about death, dying, or suicide and those who make statements such as “there’s no purpose in life” or “there’s no reason for living” and say goodbye to people as if they will not see them again or gives away cherished belongings (Kuehn, 2013; Maina et al., 2019). The nurse or midwife assesses for nonverbal signs that may potentially indicate the patient is considering self-harm, such as avoiding eye contact, tearfulness, crying, or an abrupt change in behavior, such as sudden happiness, which may signal that the patient has solidified the plan to commit suicide (Maina et al., 2019). If any of these signs are identified, it should be reported to the multidisciplinary team including a psychologist for a thorough assessment. A nurse or midwife should be assigned to such a patient for regular monitoring and care (Rudd, 2008).

Many nurses and midwives struggle with how to begin a suicide risk assessment (Tzeng et al., 2010). The nurse can start with these nine simple questions: how are you coping with what has been happening in your life? Do you ever feel like just giving up? Are you thinking about dying? Are you thinking about hurting yourself? Are you thinking about suicide? Have you thought about how you would do it? Do you know when you would do it? Do you have the means to do it? Have you ever attempted to harm yourself in the past? (Tzeng et al., 2010; Gilje et al., 2013). The nurse talks to the patient to evaluate the potential for self-injury, the history of suicide attempts by oneself or within the family to establish the risk for suicide. The questioning should be done in a non-threatening manner using simple language that the patient understands. Further probing can be done to gain an in-depth understanding of the risk for suicide and the family or significant other could provide further information as needed (Gilje et al., 2013). The need for hospitalization and safety precautions are instituted based on the findings and recommendations from the health team and family.

In addition, the nurse or midwife should appraise all possible and beneficial coping methods used by the patient including all support resources (Gilje et al., 2013). In Africa, family support is an important part of the socio-cultural context (Ugwu and De Kok, 2015) and this should be explored. In some situations, the family abandons patients with psychological problems (Tang and Qin, 2015), and this could trigger suicidal ideations, so the nurse or midwife should engage the family regularly and seek their consent and inputs for interventions instituted for the patient. In a study, Acheampong and Aziato (2018) found that family was the major reason some patients with suicidal tendencies were still alive. Participants felt committing suicide and leaving their family especially the children will expose them to the unforeseen dangers in life. Thus, the nurse or midwife should pay attention to a patient who is typically very social and interactive with family, staff, and suddenly becomes withdrawn/isolated (Maina et al., 2019). Provision should be made for the family and loved ones of the patients during care to reduce isolation and depression, which will in turn fuel suicidal ideas (Gilje et al., 2013). The presence of other people and in a nonjudgmental environment will provide opportunities for the patient to express his/her thoughts feelings which could reduce stress (Ugwu and De Kok, 2015).

In addition to the social support systems’ intervention, the nurse or midwife provides a safe environment free from sharp articles, ropes, metals, weapons, and drugs that the patient can use to harm himself/herself (Sturgeon et al., 2011). The windows and room should be well-secured and if possible, the patient should be nursed in a room at the ground floor to limit the chance of jumping from higher floors. Nurses and midwives should ensure that the patient swallows medications administered, and this should be documented. The nurse or midwife should educate the patient and family on the appropriate use of medications. Regular searching for the patient’s room is important to identify any item that can be used to commit suicide (Sturgeon et al., 2011). Comprehensive documentation of all physical care activities should be done to enhance continuity of care within the 24 h (Sturgeon et al., 2011). The nurse or midwife should discourage the patient from making decisions during severe stress and rather employ problem-solving in a constructive manner so that unforeseen access to an injurious physical object will not be used to cause self-harm (Sturgeon et al., 2011; Wade and Kitzinger, 2019).

The counseling role of nurses and midwives with the requisite skills is critical in managing patients with suicidal tendencies. Counseling will help patients to cope with stress, and this reduces suicidal ideations. Counseling provides the patient with all the important information needed to make an informed choice (Tang and Qin, 2015; Acheampong and Aziato, 2018). In view of this, there is the need to develop a strong clinical counseling unit within health care facilities and train nurses and midwives in counseling to address the psychosocial aspects of patients’ illness (Sturgeon et al., 2011). Nurses and midwives are encouraged to undertake courses in counseling, so they could offer the needed support to patients and their families even in acute care settings. Nurses and midwives could contact family members, arrange for individual and/or family crisis counseling, and link them to self-help groups and other support groups. Nurses and midwives could educate the patient on cognitive-behavioral and self-management strategies that can mitigate suicidal thoughts (Gliatto and Karani, 2016).

Within the African milieu, religion plays a vital role in coping (Currier et al., 2015). Although it has been indicated in this paper that some religious beliefs and practices negatively influence healthcare access and adherence, religiosity is used as a strong coping strategy in Africa (WHO, 2012). Some patients and families perceive prayer as a medium of communicating to God who heals, consoles, and give hope (Ugwu and De Kok, 2015). Nurses and midwives should, therefore, assess the religious needs of the patient and family and help them according to the context of care.

Nurses and midwives engaged in community level and preventive care should identify people at risk of suicide and intervene as necessary (Maina et al., 2019). The patients with suicidal tendencies who are treated and discharged should be followed up at home so any signs of relapse can be identified and treated early (Miller et al., 2017). Education of the general public on the identification of signs of suicide and ways to prevent suicide should be intensified by nurses and midwives. Again, community-level nurses and midwives should engage religious leaders and visit prayer camps to identify patients at risk and assist them to access biomedical care if they consent (Maina et al., 2019).

In all these, it is paramount for the nurse or midwife not to traverse the rights and the autonomy of patients and their families (Miller et al., 2017). Within the contemporary healthcare setting, medico-legal issues are on the rise (Rutto et al., 2012). Therefore, the authors reiterate the need to adhere to standards and policies as well as data protection laws in the quest to provide care to individuals with suicidal tendencies (Maina et al., 2019). Nurses and midwives are also not to impose their faith or religion on their patients and families even if they have rigid religious beliefs that negatively affect their health and biomedical treatment (Miller et al., 2017). Education on suicide that is simple and non-threatening, or intimidating is important to reduce the incidence and improve the health-seeking behaviors of individuals.

## Conclusion

Nurses and midwives are pivotal to healthcare delivery in Africa and globally. In the era of increasing enforcement of patients’ rights in healthcare settings, the issues of patient care that hinges the patients’ beliefs are important to discuss. Individuals with rigid religious beliefs should be cared for with open mindedness and provided with adequate information with options, so they can make their own decisions. Caring for patients with suicidal ideations can be challenging because of the seemingly unpredictable execution of their ideas. The constant observations and strict safety precautions demand a lot of effort from the multidisciplinary team including nurses and midwives. Optimal knowledge and skills of nurses and midwives on suicide should be ensured so they can contribute effectively in this area of healthcare.

## Author Contributions

LA, JP, YP, and KK gathered data and literature search, drafted the text, and contributed to the interpretation and the critical review of the draft. LA conceptualized and designed the study, supervised, coordinated, and assisted in the write up of the final manuscript. JP contributed in writing the role of the nurse in the care of patients with suicidal ideations. YP contributed in writing the spirituality and its influence of healthcare access and care. KK contributed in writing the acute and chronic health challenges with suicidal tendencies. All authors contributed to the article and approved the submitted version.

### Conflict of Interest

The authors declare that the research was conducted in the absence of any commercial or financial relationships that could be construed as a potential conflict of interest.
